# Efficacy and Safety of Recombinant Human Soluble Thrombomodulin in Patients With Sepsis-Induced Disseminated Intravascular Coagulation After Emergency Surgery

**DOI:** 10.7759/cureus.80589

**Published:** 2025-03-14

**Authors:** Keiji Nagata, Takahisa Fujikawa, Taisuke Matsuoka

**Affiliations:** 1 Surgery, Kokura Memorial Hospital, Kitakyushu, JPN

**Keywords:** anticoagulant therapy, disseminated intravascular coagulation (dic), japanese association for acute medicine (jaam) dic scores, recombinant human soluble thrombomodulin (rhstm), sepsis

## Abstract

Introduction

Recombinant human soluble thrombomodulin (rhsTM) is a therapeutic agent for sepsis-induced disseminated intravascular coagulation (DIC) and is reported to be associated with bleeding events. Although several studies on rhsTM have been reported, the safety and efficacy of rhsTM for sepsis-induced DIC after emergency laparotomy remain controversial. In this study, we aimed to investigate the efficacy, safety, and bleeding complications of rhsTM in patients with sepsis-induced DIC following emergency abdominal surgery.

Methods

In this retrospective observational study, we reviewed the data of patients who underwent emergency surgery for gastrointestinal necrosis and perforation and received rhsTM for sepsis-induced DIC at a single center between January 2014 and December 2023. We evaluated the incidence rate of bleeding complications associated with rhsTM treatment, clinical characteristics, and changes in Japanese Association for Acute Medicine (JAAM) DIC scores. Patients with DIC were identified as having the JAAM DIC diagnostic criteria (DIC score ≥4).

Results

We analyzed a total of 32 patients with sepsis-induced DIC. The APACHE II (Acute Physiology and Chronic Health Evaluation II) score at admission to the intensive care unit was 20. A total of 46.9% of the patients had poor renal function with CKD (chronic kidney disease), classified based on KDIGO (Kidney Disease: Improving Global Outcomes) stage 4 or higher, and 37.5% were on regular hemodialysis. A total of 59.4% of the patients received antithrombotic therapy. The JAAM DIC score was significantly ameliorated from the first day of rhsTM administration (5.3) to days 5-7 of rhsTM administration (3.3) (p < 0.0001). A total of 75% of the patients had a HAS-BLED (Hypertension, Abnormal Renal/Liver Function, Stroke, Bleeding History or Predisposition, Labile INR, Elderly, Drugs/Alcohol Use) score, an indicator of bleeding risk, of 3 or higher. The mortality rate in the whole cohort was 37.5%. Patients were also classified into the following groups: HAS-BLED score ≥3 (n = 24) and <3 (n = 8), and survivors (n = 20) and non-survivors (n = 12). No perioperative bleeding complications were observed.

Conclusion

rhsTM was not associated with an increased incidence of bleeding complications, even in patients with sepsis-induced DIC following emergency abdominal surgery and in critically ill patients with poor renal function or those receiving antithrombotic therapy. rhsTM is a safe and effective anticoagulant for the management of sepsis-induced DIC after emergency surgery and is clinically feasible.

## Introduction

Disseminated intravascular coagulation (DIC) is a condition characterized by excessive coagulation and vascular endothelial injury, leading to the formation of intravascular thrombin and fibrin. This results in thrombosis in small- to medium-sized vessels, causing organ dysfunction and severe bleeding [1]. In sepsis, inflammatory cytokines inhibit the expression of thrombomodulin, an anticoagulant in the vascular endothelium, and increase coagulation activation. The resulting excessive production of thrombin causes multiple microthrombi, complicating sepsis with DIC [2]. The in-hospital mortality rate for severe sepsis is still high, reported to be 20-30% [3]. DIC complicates 20-40% of sepsis cases [4, 5], and the mortality rate is reported to be even higher in patients with DIC [6, 7]. For these reasons, it is now considered a major public health problem, as it is estimated to cost more than $20 billion annually for U.S. healthcare systems [8].

Early diagnosis and early intervention are important in sepsis because the prognosis of patients who present with sepsis-induced DIC is extremely poor due to the development of multiple organ failure (MOF). Recombinant human soluble thrombomodulin (rhsTM) binds to thrombin, and then the thrombin-TM-α complex activates protein C (PC) to activated PC (APC), which inactivates factors VIIIa and Va in the presence of protein S, inhibiting further thrombin formation [9, 10]. Furthermore, the effectiveness of rhsTM in sepsis-induced DIC has been reported based on its anti-inflammatory action through the lectin-like domain [11, 12], resulting in inhibition of lipopolysaccharide (LPS) [12] and regulation of high mobility group box 1 (HMGB1) [13]. rhsTM was developed as an anticoagulant drug for sepsis-induced DIC and was approved in 2008 in Japan.

Although the anticoagulant effect of rhsTM is attenuated as thrombin levels decrease, reducing the incidence of bleeding complications, bleeding complications have been reported as an adverse event of rhsTM therapy. In addition, few studies have focused on the efficacy of rhsTM after emergency abdominal surgery and the risk of bleeding complications during rhsTM treatment [14]. Therefore, opinions are divided as to whether rhsTM should be administered promptly following emergency surgery for sepsis-induced DIC patients. In this study, we investigated the efficacy and safety of rhsTM in patients with sepsis-induced DIC following emergency abdominal surgery who were considered at high risk for bleeding complications at our hospital over the past 10 years.

## Materials and methods

**Figure 1:**
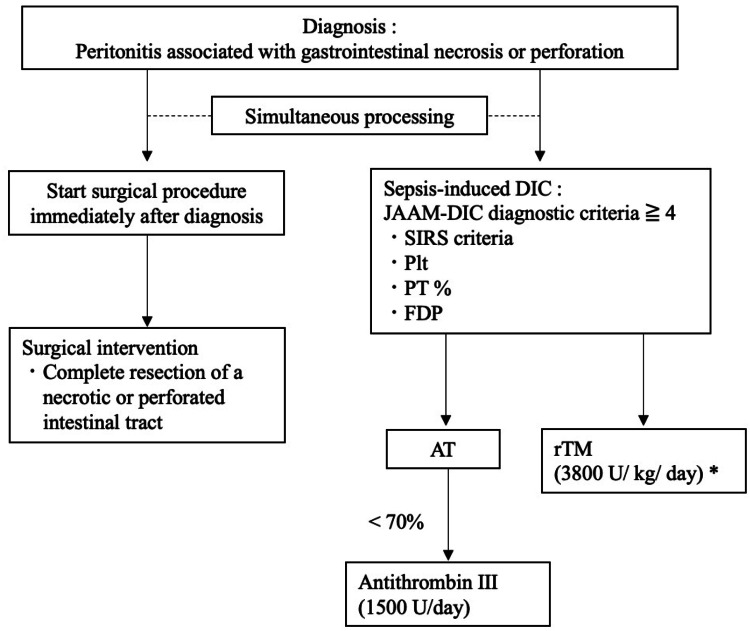
Flowchart and treatment strategy for peritonitis associated with gastrointestinal necrosis or perforation and sepsis-induced DIC *A dose of rhsTM was decreased (130 U/kg/day) in cases of severe renal dysfunction. DIC: disseminated intravascular coagulation, JAAM: Japanese Association for Acute Medicine, SIRS: systemic inflammatory response syndrome, AT: antithrombin, Plt: platelet, PT: prothrombin, FDP: fibrin/fibrinogen degradation products.

In this retrospective observational study, we analyzed the data of patients who underwent emergency surgery for gastrointestinal necrosis and perforation and received rhsTM for sepsis-induced DIC in the perioperative period at Kokura Memorial Hospital, Kitakyushu, Japan, between January 2014 and December 2023. The treatment strategy for peritonitis associated with gastrointestinal necrosis or perforation is shown in Figure 1.

We performed a surgical procedure (complete resection of a necrotic or perforated intestinal tract) immediately after diagnosis and simultaneously started treatment of sepsis-induced DIC at an early stage. The criteria for the administration of rhsTM was a diagnosis of DIC. Patients with DIC were identified using the Japanese Association for Acute Medicine (JAAM) DIC criteria (DIC score ≥ 4) [15]. The standard dose of rhsTM was 380 U/kg/day for six days. The rhsTM dose was reduced to 130 U/kg/day in cases of severe renal dysfunction. All patients were treated at the attending physician’s discretion, and no limitations were placed on the concomitant use of other anticoagulants or medications for the treatment of underlying diseases and complications. Antithrombin (AT) concentrate was administered when the AT level was below 70%. Patients with DNAR (Do Not Attempt Resuscitation) orders who died within 72 hours after treatment initiation were excluded from the study.

Data collection and definitions

A standardized evaluation of the electronic surgical database, along with hospital and clinical records, was used to collect patient characteristics, perioperative variables, and postoperative outcomes of the included patients. Bleeding complications were obtained from patients’ electronic health records. The condition of patients' functions and symptoms concerning the requirement for care and their ambulatory status were described using PS (Performance Status) and the American Society of Anesthesiologists Physical Status Classification (ASA-PS). The Acute Physiology and Chronic Health Evaluation (APACHE) II score was used as a measure of severity assessment at admission to the intensive care unit (ICU) [16]. Bleeding risk was evaluated using the HAS-BLED (Hypertension, Abnormal Renal/Liver Function, Stroke, Bleeding History or Predisposition, Labile INR, Elderly, Drugs/Alcohol Use) score, an indicator of bleeding risk [17], with a score of 3 or higher suggesting significance. Patients with DIC were identified based on the JAAM DIC diagnostic criteria (DIC score ≥4) [15]. Changes in DIC scores were measured on the first day of rhsTM administration and on days 5-7. The primary outcome of the study was the incidence rate of bleeding complications associated with rhsTM treatment. Furthermore, we evaluated these clinical characteristics and changes in JAAM DIC scores from the first day of rhsTM administration to days 5-7 of rhsTM administration between groups with HAS-BLED scores ≥3 or <3, as well as between survivors and non-survivors. The study protocol adhered to the principles outlined in the Declaration of Helsinki and received approval (#24120501) from the Kokura Memorial Hospital Clinical Research Ethics Committee.

Statistical analysis 

The categorized data were compared using the χ² (chi-square) test or Fisher’s exact probability test. Continuous variables in the patient characteristics were expressed as n (%), with the median and interquartile range. Nonparametric variables were compared using the Mann-Whitney U test or Student’s t-test. Changes in JAAM DIC scores were examined using the Wilcoxon signed-rank test. Statistical significance was set at P < 0.05. The data were statistically analyzed using JMP software version 10.0.2 (SAS Institute Inc., Cary, North Carolina).

## Results

Thirty-five patients underwent emergency surgery for gastrointestinal necrosis and perforation and received rhsTM for sepsis-induced DIC in the perioperative period during this study period. Three patients were critically ill, resulting in a policy of DNAR, and died within 72 hours of treatment initiation. These three cases were excluded. As shown in Figure 1, surgical procedures were performed immediately after the diagnosis of peritonitis associated with gastrointestinal necrosis or perforation. Simultaneously, rhsTM was administered as early as possible once DIC was diagnosed. A total of 32 patients with sepsis-induced DIC were analyzed. Patient clinical characteristics are shown in Table 1.

**Table 1:** Clinical characteristics and diagnosis of this study (n=32) PS: performance status, ASA-PS: American Society of Anesthesiologist-Physical Status, APACHE Ⅱ: Acute Physiology and Chronic Health Evaluation, ATT: antithrombotic therapy, DIC: disseminated intravascular coagulation, Intra bleeding: intraoperative bleeding, Intra-RBC Transf: intraoperative red blood cell transfusion, U: unit, AT-Ⅲ: antithrombin Ⅲ, rhsTM: recombinant human soluble thrombomodulin, CHF: chronic heart failure, CKD: chronic kidney disease, NOMI: non-occlusive mesenteric ischemia, SMA thrombosis: superior mesenteric artery thrombosis.

Factors	Values
Age, years, median (range)	73.9 (70-78)
Male gender, n (%)	20 (62.5)
PS ≥ 2, n (%)	17 (53.1)
ASA-PS ≥ 3, n (%)	29 (90.6)
APACHE Ⅱ score, median (range)	20 (14-24)
ATT, n (%)	19 (59.4)
DIC score (the first day of DIC diagnosis)	5.0 (4-6.8)
HAS-BLED score, median (range)	3 (2.3-4)
CHADS_2_ score, median (range)	3 (2-4)
CHA_2_DS_2_-VASc score, median (range)	5.5 (4.0-6.8)
Intra bleeding (mL), median (range)	60 (35-150)
Intra-RBC transf (U), median (range)	0 (0-2)
RBC transf during rhsTM therapy (U), median (range)	1 (0-2)
Use of AT-Ⅲ, n (%)	14 (43.8)
Use of heparin, n (%)	0 (0)
Duration of rhsTM therapy (day)	6 (4-6)
CHF, n (%)	18 (56.3)
CKD45, n (%)	15 (46.9)
Regular hemodialysis, n (%)	12 (37.5)
Bleeding complication, n (%)	0 (0)
Survival rate, n (%)	20 (62.5)
Duration of hospital stays (days) (range)	30 (20-71)
Diagnosis: Perforation of the stomach, n (%)	1 (3.1)
Diagnosis: Perforation of lower GI tract, n (%)	20 (62.5)
Diagnosis: NOMI, n (%)	5 (15.6)
Diagnosis: SMA thrombosis, n (%)	3 (9.4)
Diagnosis: Ischemic bowel disease, n (%)	3 (9.4)

**Figure 2:**
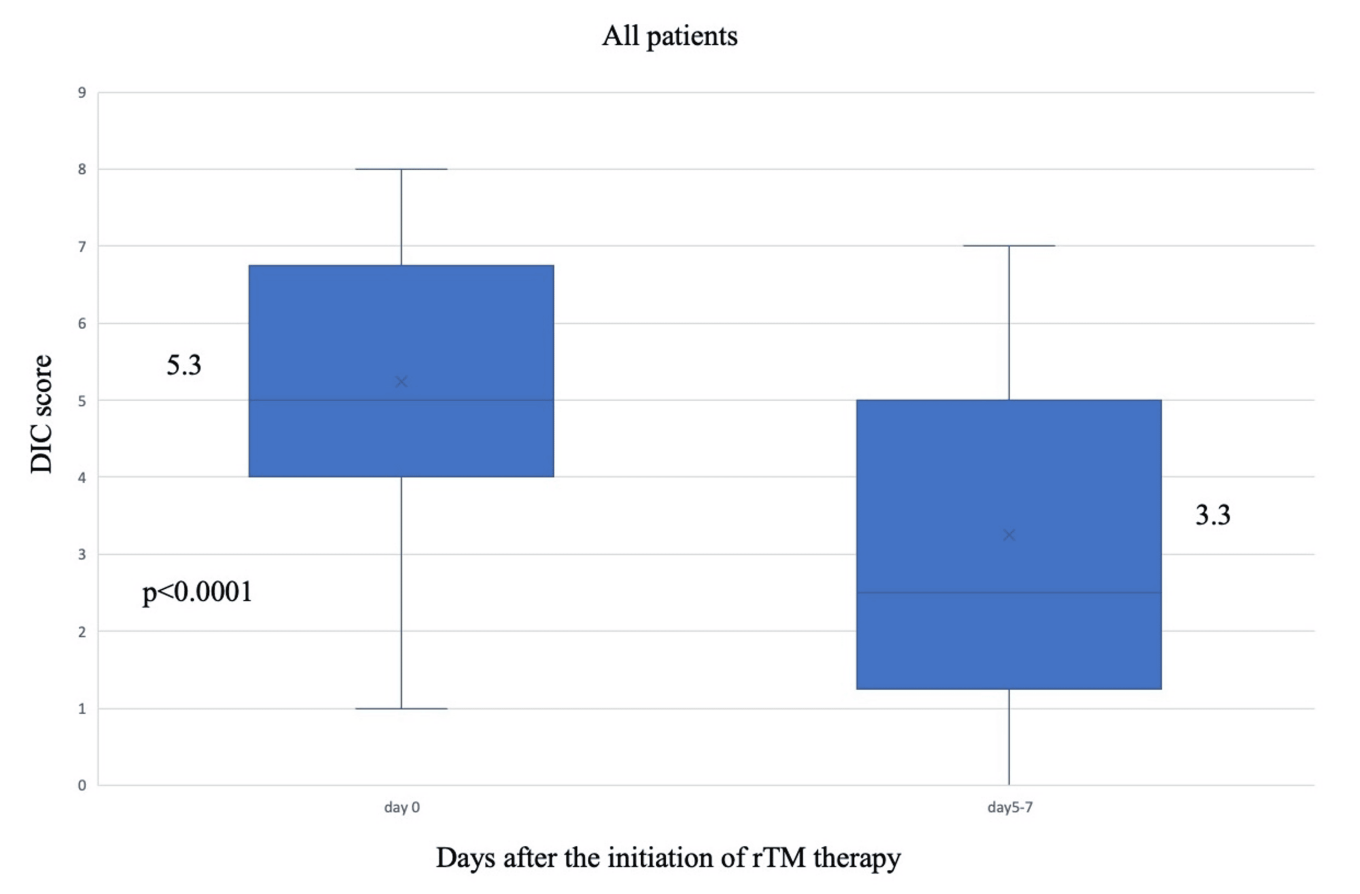
Changes of JAAM DIC score after the initiation of rhsTM therapy in all patients (n= 32) The number in the middle of each boxplot is the median value. JAAM: Japanese Association for Acute Medicine, DIC: disseminated intravascular coagulation, rhsTM: recombinant human soluble thrombomodulin.

The median age was 73.9 years, and the median APACHE II score at admission to the intensive care unit was 20, indicating high severity. The occurrences of underlying diseases, including a history of chronic heart failure and chronic kidney disease, were 56.3% and 46.9%, respectively. Regular hemodialysis (HD) was performed in 37.5% of cases. Poor PS and ASA-PS scores were observed. Diagnosed conditions included 20 cases of lower gastrointestinal tract perforation, the most common, 5 cases of non-occlusive mesenteric ischemia (NOMI), 3 cases each of superior mesenteric artery (SMA) thrombosis and ischemic bowel disease, and 1 case of upper gastrointestinal perforation. Intraoperative bleeding and red blood cell (RBC) transfusion volumes were 60 mL and 0 units, respectively, while RBC transfusion during rhsTM therapy was 1 unit. A total of 43.8% of patients received AT-III as the other anticoagulant, and no heparin was used. Antithrombotic therapy (ATT) was administered to 59.4% of patients, and 24 patients (75%) had a HAS-BLED score, an indicator of bleeding risk, of 3 or higher. No perioperative bleeding complications were observed. The survival rate in the whole cohort was 62.5%. The JAAM DIC score was significantly ameliorated from the first day of rhsTM administration to days 5-7 of rhsTM administration (P < 0.0001) (Figure 2).

**Figure 3:**
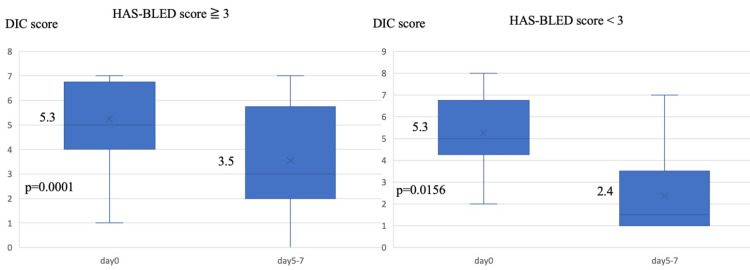
Changes in the JAAM DIC score after the initiation of rhsTM therapy in patients with a HAS-BLED score (≥3) (n = 24) and a HAS-BLED score (<3) (n = 8). The number in the middle of each boxplot is the median value. JAAM: Japanese Association for Acute Medicine, DIC: disseminated intravascular coagulation, rhsTM: recombinant human soluble thrombomodulin.

We classified these sepsis-induced DIC patients into the following groups: HAS-BLED score (≥3 (n = 24), <3 (n = 8)), survivors (n = 20), and non-survivors (n = 12). Patient backgrounds for each group are shown in Tables 2, 3. In terms of HAS-BLED score, there were no significant differences in JAAM DIC score on the first day of DIC diagnosis or in survival rate between the two groups. The HAS-BLED score (≥3) group, however, had a higher prevalence of underlying diseases, including a history of chronic kidney disease grade 4 or 5 (CKD45), and tended to have a higher percentage of regular HD. The APACHE II score was higher, and the percentage of patients who received ATT was also higher in the HAS-BLED score (≥3) group. There were no significant differences in intraoperative bleeding, intraoperative RBC transfusion, or RBC transfusion during rhsTM therapy between the two groups (Table 2). The JAAM DIC score decreased in both groups following rhsTM administration, with a significant decrease in the HAS-BLED score (≥3) and HAS-BLED score (<3) groups (P = 0.0001 and P = 0.0156, respectively) (Figure 3).

**Table 2:** Comparison between HAS-BLED score (≥3) and HAS-BLED score (<3) of this study PS: Performance Status, ASA-PS: American Society of Anesthesiologist-Physical Status, APACHE Ⅱ: Acute Physiology and Chronic Health Evaluation, ATT: antithrombotic therapy, DIC: disseminated intravascular coagulation, Intra bleeding: intraoperative bleeding, Intra-RBC Transf: intraoperative red blood cell transfusion, U: unit, AT-Ⅲ: antithrombin Ⅲ, rhsTM: recombinant human soluble thrombomodulin, CHF: chronic heart failure, CKD: chronic kidney disease, NOMI: non-occlusive mesenteric ischemia, SMA thrombosis: superior mesenteric artery thrombosis. ^#^t-values. *Chi-square value.

Factors	HAS-BLED (≥3) (n=24)	HAS-BLED (<3)	P-value	t value or chi-square value
Age, years, median (range)	73 (70-84)	71 (58-77)	0.1623	1.761^#^
Male gender, n (%)	16 (66.7)	4 (50.0)	0.3991	0.711*
PS ≥ 2, n (%)	13 (54.2)	4 (50.0)	0.8379	0.042*
ASA-PS ≥ 3, n (%)	23 (95.8)	6 (75.0)	0.0800	3.065*
APACHE Ⅱ score, median (range)	22 (16-24)	13.5 (11-20)	0.0234	2.296^#^
ATT, n (%)	17 (70.8)	2 (25.0)	0.0223	5.225*
DIC score (the first day of DIC diagnosis)	5 (4-6.8)	5 (4.3-6.8)	1.0	0^#^
CHADS_2_ score, median (range)	3 (2-4)	1.5 (0.3-2.8)	0.0067	3.264^#^
CHA_2_DS_2_-VASc score, median (range)	6 (4-7)	4 (1-5)	0.0112	3.026^#^
Intra bleeding (mL), median (range)	54 (25-135)	105 (45-324)	0.3426	0.884^#^
Intra-RBC transf (U), median (range)	0 (0-2)	0 (0-2)	0.7607	0.020^#^
RBC transf during rhsTM therapy (U), median (range)	2 (0-3.5)	0 (0-1.5)	0.0714	2.5204^#^
Use of AT-Ⅲ, n (%)	9 (37.5)	5 (62.5)	0.2170	1.524*
Use of heparin, n (%)	0 (0)	0 (0)	-	-
Duration of rhsTM therapy (day)	5.5 (4-6.8)	6 (3.5-6)	0.8586	0.4367^#^
CHF, n (%)	15 (62.5)	3 (37.5)	0.2170	1.524*
CKD45, n (%)	14 (58.3)	1 (12.5)	0.0245	5.061*
Regular hemodialysis, n (%)	11 (45.8)	1 (12.5)	0.0917	2.844*
Bleeding complication, n (%)	0 (0)	0 (0)	-	-
Survival rate, n (%)	13 (54.7)	7 (87.5)	0.0917	2.844*
Duration of hospital stays (days) (range)	29 (21-63)	49 (11-84)	0.8789	0.6436^#^

**Figure 4:**
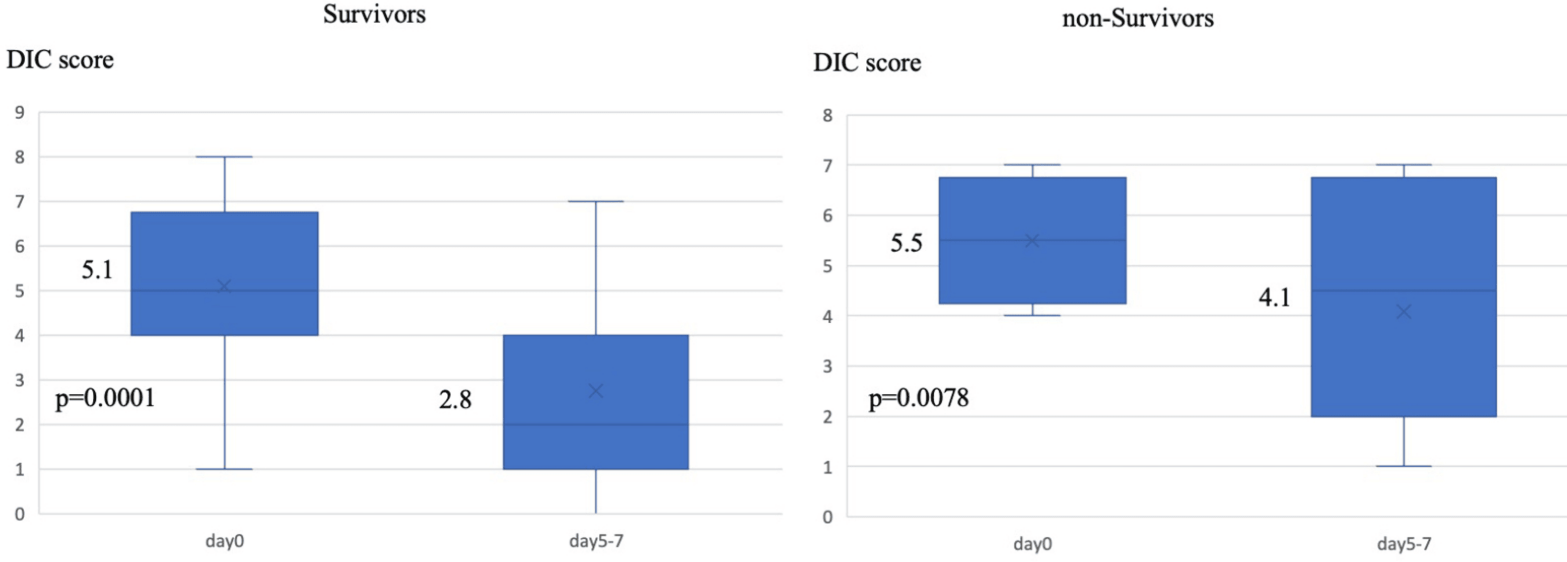
Changes in the JAAM DIC score after the initiation of rhsTM therapy in survivors (n = 20) and non-survivors (n = 12) The number in the middle of each boxplot is the median value. JAAM: Japanese Association for Acute Medicine, DIC: disseminated intravascular coagulation, rhsTM: recombinant human soluble thrombomodulin.

In terms of survival, there were no significant differences in JAAM DIC score on the first day of DIC diagnosis or in the percentage of patients who received ATT between the two groups. The non-survivor group, however, had a higher proportion of patients with a HAS-BLED score (≥3) and tended to have a higher prevalence of underlying diseases, including a history of CKD45 and regular HD. There were no significant differences in intraoperative bleeding, intraoperative RBC transfusion, or RBC transfusion during rhsTM therapy between the two groups (Table 3). The JAAM DIC score decreased in both groups following rhsTM administration, with a significant decrease in survivors and non-survivors (P = 0.0001 and P = 0.0078, respectively) (Figure 4).

**Table 3:** Comparison between survivors and non-survivors of this study. PS: performance status, ASA-PS: American Society of Anesthesiologist-Physical Status, APACHE Ⅱ: Acute Physiology and Chronic Health Evaluation, ATT: antithrombotic therapy, DIC: disseminated intravascular coagulation, Intra bleeding: intraoperative bleeding, Intra-RBC Transf: intraoperative red blood cell transfusion, U: unit, AT-Ⅲ: antithrombin Ⅲ, rhsTM: recombinant human thrombomodulin, CHF: chronic heart failure, CKD: chronic kidney disease, NOMI: non-occlusive mesenteric ischemia, SMA thrombosis: superior mesenteric artery thrombosis. ^#^t-value. *Chi-square value.

Factors	Survivors (n=20)	Non-Survivors (n=12)	P-value	t-value or chi-square value
Age, years, median (range)	73 (68-86)	74 (70-78)	0.8759	0.1864^#^
Male gender, n (%)	10 (50.0)	10 (83.3)	0.0593	3.556*
PS ≥ 2, n (%)	11 (55.0)	6 (50.0)	0.7838	0.075*
ASA-PS ≥ 3, n (%)	18 (90.0)	11 (91.7)	0.8756	0.025*
APACHE Ⅱ score, median (range)	18 (12.5-23.8)	21.5 (17-23.8)	0.3393	0.938^#^
ATT, n (%)	10 (50)	9 (75)	0.1633	1.943*
DIC score (the first day of DIC diagnosis)	5 (4-6.8)	5.5 (4.3-6.8)	0.6327	0.694^#^
HAS-BLED score, median (range)	3 (2-3.8)	4 (3-5)	0.0200	2.322^#^
CHADS_2_ score, median (range)	2.5 (1-3.8)	3.5 (2.3-4)	0.0448	2.300^#^
CHA_2_DS_2_-VASc score, median (range)	5 (3-6)	6 (5-7)	0.1391	1.790^#^
Intra bleeding (mL), median (range)	54 (35-200)	65 (17-146)	0.9031	0.208^#^
Intra-RBC transf (U), median (range)	0 (0-2)	0 (0-2)	0.5233	0.291^#^
RBC transf during rhsTM therapy (U), median (range)	0 (0-2)	2 (0-4)	0.2348	1.369^#^
Use of AT-Ⅲ, n (%)	8 (40)	6 (50)	0.5809	0.305*
Use of heparin, n (%)	0 (0)	0 (0)	-	-
Duration of rhsTM therapy (day)	5 (4-6)	6 (4.5.7)	0.1204	1.092^#^
CHF, n (%)	10 (50)	8 (66.7)	0.3575	0.847*
CKD45, n (%)	7 (35)	8 (66.7)	0.0822	3.020*
Regular hemodialysis, n (%)	5 (25)	7 (58.3)	0.0593	3.556*
Bleeding complication, n (%)	0 (0)	0 (0)	-	-
Duration of hospital stays (days) (range)	37 (22-78)	23 (18-54)	0.3400	0.7016^#^

## Discussion

We investigated the efficacy and safety of rhsTM retrospectively in patients with sepsis-induced DIC following emergency abdominal surgery who were at particularly high risk of bleeding complications. In this study, rhsTM was used immediately after emergency abdominal surgery for sepsis-induced DIC. Furthermore, even in critically ill patients with elevated APACHE II scores, poor renal function, or those receiving ATT, rhsTM improved sepsis-induced DIC without increasing bleeding complications.

A meta-analysis evaluating the efficacy and safety of rhsTM in patients with sepsis-induced DIC, which included 11 studies, reported that the incidence of bleeding complications in patients treated with rhsTM was 8.8% [18]. Onoda et al. [19] examined bleeding complications according to renal function classification, based on the Kidney Disease: Improving Global Outcomes (KDIGO) 2012 Clinical Practice Guideline for the Evaluation and Management of Chronic Kidney Disease [20]. Patients were classified into six groups: G1, G2, G3a, G3b, G4, and G5 (G4 was 30 mL/min/1.73 m^2^ > eGFR ≥ 15 mL/min/1.73 m^2^; and G5 was eGFR < 15 mL/min/1.73 m^2^, respectively). In this study, he reported that the incidence of bleeding complications following rhsTM treatment in the G4 and G5 groups was 11.1% and 60.0%, respectively, with adverse bleeding complications observed particularly in the G5 group. He suggested that the overall incidence rate of bleeding complications differed significantly by renal function and that bleeding events tended to increase with the deterioration of renal function. In this study, 46.9% of the patients had poor renal function with CKD classified as KDIGO stage 4 or higher, and 37.5% of the patients underwent regular HD. Although many patients in this study had poor renal function, no bleeding complications occurred in the whole cohort.

Furthermore, we used the HAS-BLED score to investigate bleeding complications in the present study. The HAS-BLED score predicts the risk of bleeding in patients receiving anticoagulation therapy (ACT), as it is well known that ACT is associated with a risk of bleeding, and HAS-BLED scores of 3 or higher indicate high-risk patients for bleeding complications [17]. Since 59.4% of patients in this study received ATT, which included antiplatelet therapy (APT) and/or ACT, we used the HAS-BLED score to assess bleeding risk. To the best of our knowledge, there are no studies assessing bleeding complications using the HAS-BLED score in patients who underwent emergency abdominal surgery and received rhsTM for sepsis-induced DIC. Although 75% of patients had a HAS-BLED score of 3 or higher, there was no difference in mortality rates, intraoperative bleeding, intraoperative RBC transfusion, or RBC transfusion during rhsTM therapy between the groups with a HAS-BLED score (≥3) and <3. No bleeding complications occurred in the whole cohort.

Sepsis-induced DIC is a poor prognosis condition with severe ischemic organ damage and an extremely high mortality rate of 34.7% [7]. Lower gastrointestinal tract perforation, NOMI, SMA thrombosis, and ischemic bowel disease are conditions of peritonitis with gastrointestinal necrosis or perforation that require immediate surgical intervention. The prognosis is even worse for DIC associated with peritonitis and such gastrointestinal necrosis and perforation. Anticoagulation therapy for sepsis-induced DIC is crucial, alongside addressing the underlying disease, with rhsTM being one of the anticoagulants. Two meta-analyses have demonstrated the efficacy of rhsTM in sepsis-associated coagulopathy [21, 22]. Additionally, a phase III randomized, double-blinded study indicated that rhsTM significantly improved the resolution rate of DIC and reduced 28-day mortality in patients with DIC-associated infectious diseases [10]. On the other hand, a large randomized clinical trial (SCARLET trial) reported no reduction in 28-day mortality when rhsTM was used to treat sepsis-induced coagulopathy [23].

This study demonstrates a significant reduction in the JAAM DIC score from the first day of rhsTM administration to days 5-7 (P < 0.0001) in both survival and non-survival groups following rhsTM administration. Notably, the JAAM DIC score further decreased on days 5-7 of rhsTM administration, specifically in the survivor group. The timing of DIC improvement with rhsTM varies among reports, potentially due to differences in patient backgrounds, such as disease severity. Various biomarkers of inflammation have been proposed for early and specific diagnosis of systemic inflammation and sepsis. Lower levels of butyrylcholinesterase (BChE), a non-specific cholinesterase enzyme, have been correlated with infectious diseases and septic shock, with ongoing research into the utility of BChE in multiple systemic inflammatory conditions [24]. The use of such a marker may aid in the early diagnosis of sepsis and possibly facilitate early treatment in critically ill patients.

Limitation

This study had several limitations. First, this study had a retrospective observational design and was conducted at a single institution with an insufficient number of cases. This reduces the significance of the findings. Second, this study was not a randomized, placebo-controlled trial, which may limit the generalizability of our findings. Third, all patients were treated according to the attending physicians’ decisions, and there may be confounding factors between rhsTM administration and perioperative complications. Fourth, this study does not compare DIC outcomes with those of non-rhsTM patients and thus may not accurately reflect the effects of rhsTM. Fifth, the causes of bleeding are complex, making it difficult to determine whether the bleeding is exclusively attributable to rhsTM. Future studies are required to collect and analyze more cases in order to determine the efficacy and safety of rhsTM in patients with sepsis-induced DIC following emergency abdominal surgery.

## Conclusions

In our study, rhsTM was not associated with an increased incidence of bleeding complications, even in patients with sepsis-induced DIC following emergency abdominal surgery who were at particularly high risk of bleeding complications, including critically ill patients with poor renal function or those receiving ATT. rhsTM is a safe and effective anticoagulant for the management of sepsis-induced DIC. Further studies are required to clarify the efficacy and safety of rhsTM in patients with sepsis-induced DIC.
